# Optical Coherence Tomography Angiography of the Intestine: How to Prevent Motion Artifacts in Open and Laparoscopic Surgery?

**DOI:** 10.3390/life13030705

**Published:** 2023-03-06

**Authors:** Maksim Ryabkov, Mikhail Sizov, Evgeniya Bederina, Polina Zarubenko, Peter Peretyagin, Alexander Moiseev, Aleksander Vorobiev, Natalia Gladkova, Vladimir Zaitsev, Elena Kiseleva

**Affiliations:** 1 University Clinic, Privolzhsky Research Medical University, 18/1 Verkhnevolzhskaya Naberezhnaja, 603155 Nizhny Novgorod, Russia; 2 City Clinical Hospital No.30, 85A Berezovskaya St., 605157 Nizhny Novgorod, Russia; 3 Department of General, Operative Surgery and Topographic Anatomy Named after A.I. Kozhevnikov, Privolzhsky Research Medical University, 190 Rodionova St., 603093 Nizhny Novgorod, Russia; 4 Central Research Laboratory, Privolzhsky Research Medical University, 70 Gagarin Ave., 603081 Nizhny Novgorod, Russia; 5 Institute of Applied Physics of the RAS, 46 Ulyanova St., 603950 Nizhny Novgorod, Russia; 6 Institute of Experimental Oncology and Biomedical Technologies, Privolzhsky Research Medical University, 10/1 Minin and Pozharsky Sq., 603005 Nizhny Novgorod, Russia

**Keywords:** optical coherence tomography angiography (OCTA), motion artifacts, intestine, blood vessels, microvessels, ischemia, intraoperative diagnostics, vacuum stabilized imaging

## Abstract

(1) Introduction. The problem that limits the intraoperative use of OCTA for the intestinal circulation diagnostics is the low informative value of OCTA images containing too many motion artifacts. The aim of this study is to evaluate the efficiency and safety of the developed unit for the prevention of the appearance of motion artifacts in the OCTA images of the intestine in both open and laparoscopic surgery in the experiment; (2) Methods. A high-speed spectral-domain multimodal optical coherence tomograph (IAP RAS, Russia) operating at a wavelength of 1310 nm with a spectral width of 100 μm and a power of 2 mW was used. The developed unit was tested in two groups of experimental animals—on minipigs (group I, n = 10, open abdomen) and on rabbits (group II, n = 10, laparoscopy). Acute mesenteric ischemia was modeled and then 1 h later the small intestine underwent OCTA evaluation. A total of 400 OCTA images of the intact and ischemic small intestine were obtained and analyzed. The quality of the obtained OCTA images was evaluated based on the score proposed in 2020 by the group of Magnin M. (3) Results. Without stabilization, OCTA images of the intestine tissues were informative only in 32–44% of cases in open surgery and in 14–22% of cases in laparoscopic surgery. A vacuum bowel stabilizer with a pressure deficit of 22–25 mm Hg significantly reduced the number of motion artifacts. As a result, the proportion of informative OCTA images in open surgery increased up to 86.5% (Χ^2^ = 200.2, *p* = 0.001), and in laparoscopy up to 60% (Χ^2^ = 148.3, *p* = 0.001). (4) Conclusions. The used vacuum tissue stabilizer enabled a significant increase in the proportion of informative OCTA images by significantly reducing the motion artifacts.

## 1. Introduction

The correct determination of bowel resection borders, based on objective data on its blood supply, allows for avoiding the erroneous over- or underestimation of the operation volume. In turn, the optimal choice of the bowel resection volume is the main mechanism for the reduction of the risk of postoperative complications including secondary ischemia and necrosis of the intestine, anastomotic failure, and short bowel syndrome [1]. In contrast to the preoperative diagnosis, the intraoperative examination of intestinal circulation is associated with the need for a multiple targeted visualization of the vascular network in small, compromised areas of the intestine. Therefore, the efficiency of technologies of intraoperative diagnostics of intestinal blood flow will be determined by such factors as the possibility of multiple contrast-agent-free examinations with a short period of time needed for tissue assessing (from several seconds to several minutes), the collection of the data directly during the surgery without additional image processing, scanning depth in the range of 1–2 mm, and the ability of integration into laparoscopic instruments. Conventional technologies of radiation diagnostics (computer tomography and magnetic resonance imaging) do not meet the aforementioned requirements. The main disadvantage is their large size that is related to the use of large lenses and rigid filters that prevent their miniaturization and integration into surgical instruments [2]. 

A number of bioimaging technologies allow for non-contrast diagnostics during abdominal surgery (for example, Laser speckle-contrast imaging (LSCI), sidestream dark field imaging or incident dark field imaging). However, they do not have either sufficient scanning depth or resolution to visualize intramural vessels in the layers of the intestinal wall. In addition, the quality of LSCI and sidestream dark field imaging images is critically reduced by the motions of the object under study [3]. Fluorescein angiography requires the injection of a special dye into the bloodstream each time before the study. This increases the manipulation time and virtually eliminates multiple revisions of individual tissue sections. Some new technologies that have sufficient resolution and do not require the introduction of contrast agents into the bloodstream (for example, sidestream dark field imaging and incident dark field imaging, hyperspectral imaging) are capable of visualizing only superficial vessels of the serous membrane of the intestine with transabdominal access [4,5]. Like most high-resolution bioimaging methods, dark-field microscopy is very sensitive to the motions of the object under study in vivo and requires additional tissue immobilization to obtain informative images [6]. To summarize, despite the active development of bioimaging technologies, the need for clinicians for intraoperative non-contrast label-free diagnostics of intramural circulation in the small intestine has not yet been satisfied [7].

Optical coherence tomography angiography (OCTA) has the necessary potential for the objective assessment of intestinal circulation in emergency surgery. Experimental studies of small laboratory animals have confirmed the sufficient resolution for the visualization of intestinal intramural vessels with a diameter of 10 to 1000 microns; the scanning depth was sufficient to visualize all layers of the intestinal wall (1.5–2 mm), and there was an acceptable data acquisition speed (20 s per OCTA image). The efficiency of OCTA diagnostics of the digestive tract has been confirmed by several pilot clinical studies. It has been established that OCTA can provide a visualization of human blood vessels in the serosa, muscle and submucosal layers and in the mucous membrane [8]. In a transluminal study in humans, OCTA allows for assessing the blood supply to the sections of the digestive tract accessible to the endoscope—the esophagus and colon [9,10]. However, despite its high potential, optical coherence angiography has not yet become the “gold standard” for diagnosing normal and impaired blood circulation in the intestinal wall.

The problem that limits the use of OCTA for diagnosing intestinal circulation is the high proportion of non-informative OCTA images with too strong artifacts. The reason for the artifact’s appearance is the displacement of the diagnostic object relative to the OCT probe [11]. It is important that the risk of obtaining non-informative OCTA for some organs, including the intestine, increases with the size of the organism under study. When examining the intestines of laboratory rats, the number of artifacts on OCTA images is minimal, while in humans and large laboratory animals (for example, minipigs) it is unacceptably high [12]. Artifacts of movement on the intestinal OCTA are a consequence of peristalsis, transmission vibrations from the thoracic diaphragm, and the aorta. As a result of their appearance, the proportion of non-informative OCTA images in large animals (in the experiment) and in humans (in the clinic) reaches 50% [13].

None of the currently proposed methods for preventing movement artefacts has made it possible to fundamentally increase the information content of the intestinal OCTA. To solve this problem, the mechanical pressing of the probe to the tissue under study is used. However, the anatomical and physiological features of the small intestine of large animals and humans (peristaltic contractions, displacement of intestinal segments within the abdominal cavity, wet sliding surface of the serous membrane, lack of dense underlying tissues to which one could “press” the intestine in the abdomen) critically reduce the effectiveness of this technique. A fundamentally different approach to solving the problem is based on the digital post-processing of the obtained OCTA images, for example, a method of numerical compensation for the influence of longitudinal mutual displacements of the probe and tissue or multiple wavelet filtering and reconstruction [14]. However, the improvement in the quality of OCTA images in this case is provided due to additional data processing, which limits the use of the method in the real conditions of an emergency operation. Some of the digital methods used to compensate for artifacts of the OCTA image may themselves have undesirable consequences for image quality [15]. A reduction in intraoperative time to image acquisition and a significant reduction in the number of artefacts can be obtained in the case of scanning in M-mode [16]. However, these effects are achieved at the cost of refusing to obtain a 3D OCTA picture, which reduces the information content of the data. 

Machine learning and neural networks are widely used to process and classify huge amounts of data, including the application to various kinds of images enabled by OCT. For example, some studies relate to the “digital staining” of OCT scans in order to obtain images similar to conventional stained histological slides [17,18], whereas others consider the possibilities of the segmentation of various morphological components directly in structural OCT scans (e.g., [19,20]. Concerning the functional extensions of OCT, machine learning approaches are considered for reconstruction and improving the quality of angiographic OCT images [21,22]. Additionally, similarly to the analysis/segmentation of structural OCT scans, machine-learning methods are considered for the segmentation/analysis of OCTA images, in particular, for automated revealing zones of perturbed microcirculation [23,24,25,26]. Quite a comprehensive overview of various aspects of machine-learning-based studies related to OCTA can be found in the recent paper [27]. Potentially, such algorithms could be well suited for automated determining artefacts in OCTA images (and their suppression where possible), as well as for estimating the percentage of artefacts that cannot be removed to reject OCTA data of insufficient quality unsuitable for analysis (e.g., [28,29,30]. Certainly, the automated rejection of the insufficient quality of OCTA images is a very useful possibility, however, for practical clinical applications, a critically important problem is the reduction of the percentage of insufficient-quality data. Indeed, the available time for intraoperative OCTA examinations is very limited, so that repeated OCTA examinations for gathering images of sufficient quality are often impossible. The post-processing of OCTA images (even using the most sophisticated algorithms) cannot help if the initially acquired data are too strongly degraded. In view of this, the problem of enhancing the quality of the initial gathering of OCTA data is of utmost importance for the translation of OCTA to real clinical applications. However, an effective and affordable technology that reduces the proportion of artefacts in OCTA images and allows for a significant increase in the quality of intraoperative diagnostics in large animals and humans has not yet been developed.

Robotic and laparoscopic operations further complicate the task of obtaining high-quality images in intestinal OCTA. The laparoscopic diagnosis of the spread and depth of ischemia in the intestine is a mandatory procedure in accordance with international, European and many national clinical guidelines [7]. During laparoscopic operations, the surgeon’s tactile, visual sensations are distorted by “mediators”—instruments and color rendering of the video system. Additional objective information about the state of intramural circulation is especially in demand in such conditions. Attempts are being made to adapt the LSCI, hyperspectral imaging, OCT technologies to the conditions of laparoscopic surgery, but due to the imbalance of costs and the information content of the data obtained, these technologies have not yet found wide application in practical surgery [1,31,32]. Thus, the problem of objectifying intraoperative data on blood circulation in the small intestine has not yet been solved.

Despite the experimental confirmation of the OCTA efficiency in diagnosing intestinal ischemia, the problem of the low quality of OCTA images obtained in intraoperative conditions in the clinic remains unresolved. The existing methods for reducing the number of artefacts in OCTA images are not suitable for use in a real emergency operation, since they either increase the time of data processing or reduce the amount of information received. The low informativeness of the OCTA does not allow for differentiating the parts of the intestine with normal and impaired blood circulation, making OCTA inappropriate for the clinical diagnostics of blood circulation in the small intestine. At the same time, data have been published on the efficiency of devices—stabilizers of the tissue under study in dark-field microscopy, the operation of which is based on a local pressure deficit (vacuum) [16,33]. Previously published data on the use of such devices are limited by the conditions of open (not laparoscopic) surgery [16]. 

Here, we report the first results of the practical application of a vacuum device to stabilize the intestinal wall during intraoperative OCT application in open and laparoscopic bowel surgery. We describe the development of a simple but effective technology that can be easily applicable in a clinical setting—a tissue stabilizing unit: from its design to determining the conditions for its safe and effective use in open and laparoscopic abdominal surgeries. The purpose of this study was to evaluate the efficacy and safety of the developed unit for preventing motion artifacts in OCTA images of the intestine in open and laparoscopic surgery in large laboratory animals.

## 2. Materials and Methods

### 2.1. Vacuum Tissue Stabilizer

**Figure 1:**
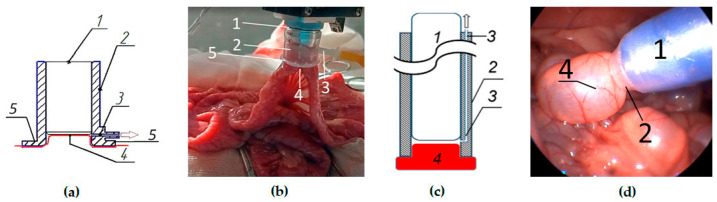
This is a figure. Schemes follow the same formatting. Vacuum tissue stabilizer during OCTA examination. (**a**)—scheme of the unit for open surgery; (**b**)—photo of the vacuum unit during OCTA of the small intestine in the open abdomen; (**c**)—scheme of the unit for laparoscopic surgery; (**d**)—photo of the vacuum unit during OCTA of the small intestine in a laparoscopic operation. 1—OCTA probe, 2—branch pipe wall, 3—aspiration channel, 4—the examined intestine tissue, 5—protective pads on the terminal part of the branch pipe of the unit for operations on the open abdomen.

For open abdominal operations, the constructed unit is a 2 cm long branch pipe made of transparent plastic, which is hermetically installed on the terminal section of the OCT probe. The branch pipe is connected to an aspirator to create a pressure deficit around the working part of the OCT probe. Due to the pressure deficit, the examined area of the intestine is pressed and fixed to the surface of the OCT probe (Figure 1a,b).

The modified vacuum unit for laparoscopic surgery is a longer branch pipe (from 12 to 20 cm to correspond to the length of the distal end of the OCT probe) with an intra-wall channel for aspiration. The branch pipe is hermetically put on the OCTA probe with an elongated terminal section, and then the probe is inserted into the abdominal cavity through an endoscopic port with a diameter of 15 mm. The proximal opening of the aspiration channel should be located outside the abdominal cavity in order to connect the aspirator tube to it. The distal opening is located in the abdominal cavity in the area of contact between the terminal section of the OCT probe and the studied intestine. The aspirator creates a negative pressure in the distal opening of the channel, and the tissue is pressed against the surface of the probe (Figure 1c,d).

### 2.2. Experimental Study Design

The device was tested in two series of experiments—on minipigs (group I, n = 10) and on rabbits (group II, n = 10). The animals were kept in accordance with the rules adopted by the European Convention for the Protection of Vertebrate Animals used for Experimental and Other Scientific Purposes (Strasbourg, 1986). The experimental study was approved by the Ethics Committee of the Privolzhsky Research Medical University (Protocol #12 from 8 August 2022). Minipigs and rabbits were chosen for experiments due to the proximity to the anatomical and physiological parameters of the human digestive tract and the possibility of open and laparoscopic surgery with control of hemodynamic parameters (monitor uMEC12 Vet Mindray).

**Figure 2:**
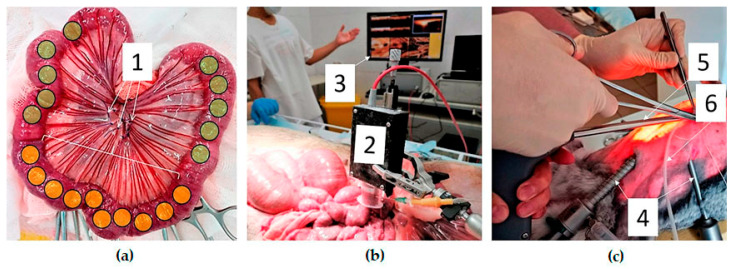
Experimental OCTA study. (**a**)—group I, open abdomen surgery, ischemic segment of the intestine taken out through the laparotomy wound (1—mesenteric arteries are ligated, yellow marks—areas of the OCTA study of the ischemic intestine, green marks—areas of the OCTA study of the intact intestine); (**b**)—group I, intraoperative OCTA using vacuum tissue compression (2—OCTA probe with the connected vacuum unit, 3—monitor with displayed OCT/OCTA images); (**c**)—group II, OCTA during laparoscopic surgery (4—instruments and a camera inserted into the abdominal cavity, 5—OCTA probe, 6—suction tube).

The first series of experiments was carried out on mini-pigs mini-sibs weighing 25–42 kg, age 4–5 months. Mesenteric bowel ischemia was modeled in open surgery. After general anesthesia with mechanical ventilation, the animals underwent median laparotomy. A section of the jejunum 40–50 cm long was removed into the laparotomy wound and normal blood circulation in the intestine wall was studied using OCTA. Acute occlusive mesenteric ischemia was modeled by the ligation of three aa. jejunales (branches of a. mesenterica cranialis) (Figure 2).

In the area of 20–25 cm of the jejunum, the inflow of arterial blood through the directly supplying arteries stopped, but partial blood supply through the intramural collateral pathways remained. After ligation of the arteries, the ischemic bowel loop was immersed into the abdominal cavity and, after 120 min, was again removed for a control OCTA study of blood circulation. Studied sections of the intestine were excised at the end of the operation and their histological examination was performed.

The second series of experiments (Figure 2c) was performed on Chinchilla rabbits. After anesthesia, conditions of laparoscopic surgery were modeled. A pneumoperitoneum was applied through a Veress needle, a laparoscope and instruments were introduced into the abdominal cavity, and clips were applied to aa. jejunales to a 15–20 cm section of the jejunum to model the occlusive ischemia. At the final stage of the experiment, a laparotomy was performed; the examined sections of the intestine were excised for histological evaluation.

In both series of experiments, OCTA of the normal and ischemic intestine was performed. For each animal, 20 OCTA images were obtained: 5 OCTA images on the ischemic area and 5 OCTA images on the normal tissues, each time using two methods of the examination: first, the manually supported contact and then using the tissue stabilizer. Thus, both in the first and second series of experiments, a total of 200 OCTA images were obtained: 4 groups of 50 images of each type.

### 2.3. Device Security Control

In the security context, the principal task was to determine a safe level of pressure deficit, which allows for fixing the intestine without a significant disturbance of blood circulation. It is known from previous studies that blood circulation in the intestinal wall remains sufficient if the perfusion pressure (PP) is maintained above 60 mm Hg. PP is a calculated indicator: it is the difference between mean arterial pressure (MAP) and intra-abdominal pressure (IAP).

MAP was calculated using the formula:

MAP = systolic blood pressure + 2 × diastolic blood pressure/3

The value of IAP in the open abdomen was considered to be equal to “0” (equal to atmospheric pressure). In laparoscopic surgery, the IAP value was determined according to the readings of the insufflator manometer.

PP was calculated by the formula:

PP = MAP − IAP

To maintain a safe level of PP (at least 60 mm Hg), the allowable pressure deficit (PD) in the device was calculated using the following formula:

PD (mm Hg) < PP (mm Hg) − 60 (mm Hg),

where PD is the permissible level of negative pressure in the lumen of the vacuum stabilizer.

### 2.4. Histological Examination

At the end of the experiment, regions of interest of the intestinal wall studied with OCTA were excised and taken for blinded histological evaluation. Tissues were fixed in 10% formalin, sent to standard histological wiring, and embedded in paraffin blocks (HistoStar, Thermo Scientific, Waltham, MA, USA). Serial histological sections 4–6 microns thick (Microm HM 325 microtome, Thermo Scientific) were stained with hematoxylin and eosin (Gemini AS, Thermo Scientific). The presence of hemorrhages and mechanical ruptures in the layers of the intestinal wall which could be the result of using a vacuum stabilizer were assessed by an independent histopathologist.

### 2.5. OCTA Device and Blood Vessels Quantification

The OCTA method is described in detail in [8,12,13]. A high-speed spectral multimodal optical coherence tomograph with an angiography mode (Institute of Applied Physics of RAS, Nizhny Novgorod, Russia) operating at a wavelength of 1310 nm, with a spectral width of 100 μm, and a power of 2 mW was used [34,35]. The longitudinal resolution is 10 µm, the depth resolution is 15 µm, the scanning depth in air is ~1.7 mm, and the scanning speed is 20,000 A-scans per second. The OCTA device is equipped with a flexible fiber optic probe, which ends with a detachable end lens with an external diameter of 8 mm. The scanning of the intestinal tissue was carried out by the contact method and the recording of one data volume (2.4 × 2.4 × 1.3 mm) took 26 s. The picture of blood vessels network is visualized during the scanning. High-frequency filtering is used to recognize moving scatters against the background of motionless tissue structures. As a result, areas with the presence of blood flow (the movement of red blood cells) are visualized as bright spots, whereas regions where the blood is in a stationary state remained dark. The smallest vessel diameter that can be distinguished is 15 µm.

The quantification of blood vessels included the calculation of the total length of the perfused vessels (L, mm) and was performed in the Python 3.6 program. Additionally, the lengths of blood vessels with a small diameter (<30 µm corresponding to capillaries and post-/pre-capillaries), medium ones (30–65 µm, arterioles and venules), and a large diameter (>65 µm, arterioles/venules and arteries/veins) were calculated [36]. A division by diameters was chosen to see which components of the microvasculature are distorted by motion artifacts to a higher extent.

The stages of automated post processing included binarization, skeletonization (finding the center line of each blood vessel), followed by the summation of all pixels responsible for the skeleton of blood vessels and converting the obtained values to millimeters.

### 2.6. Quality Evaluation of the OCTA Images

The quality of the obtained OCTA images was assessed on the basis of a modified score proposed by Magnin M. et al. (2020) to analyze the quality of microvasculature images [37]. An independent expert classified each of the obtained OCTA images into one of the classes in accordance with the following scale: class 1—artifact-free images of excellent quality; grade 2—images with a small (no more than 2) number of artifacts that make diagnosis difficult; grade 3—non-informative OCTA images with a large (more than 2) number of artifacts.

### 2.7. Statistical Data Processing

For statistical data processing the program, IBM SPSS Statistics software, V20 (IBM Corporation, Somers, NY, USA) was used. The results were expressed as the Me [Q1; Q3] where Me is the median value of the analyzed parameters (length of blood vessels in OCTA images, mm) and [Q1; Q3] are the 25th and 75th percentile values, respectively. To compare qualitative indicators, Fisher’s exact two-tailed test and Χ^2^ criterion were used. The Wilcoxon test was used to assess the statistical significance of the differences between the quantitative values obtained before and after the use of the vacuum stabilizer. The critical value of the significance level was taken equal to 5% (*p* ≤ 0.05).

## 3. Results

### 3.1. OCTA Image Quality in Open Surgery

Evaluation of the OCTA image quality of the small intestine in the conditions of the “open abdomen” showed that the application of the vacuum tissue stabilizer resulted in a statistically significant increase in the proportion of informative images of classes 1 and 2. With the manual method of the OCT probe fixation, artefact-free OCTA images of class 1 were obtained only in 14 observations out of 200; images with a small number of artifacts that still allowed one to perform diagnostics (class 2) amounted to 74 images out of 200; more than a half (112 out of 200) of the OCTA images were uninformative because of the large number of motion artifacts. In the study of the ischemic intestine, the quality of the obtained data was even lower than in the study of the intact intestine: the total number of non-informative images was 136, class 1 OCTA images were obtained in eight cases (Table 1). At the same time, the study of the ischemic small intestine is associated with a greater number of artefacts on OCTA images than the study of the intact intestine (*p* = 0.030): in the intact intestine, the proportion of non-informative images of class 3 was 112 out of 200, in the ischemic one—136 out of 200. The spasm of the intestine during prolonged ischemia creates additional difficulties for obtaining high-quality OCTA data.

**Figure 3:**
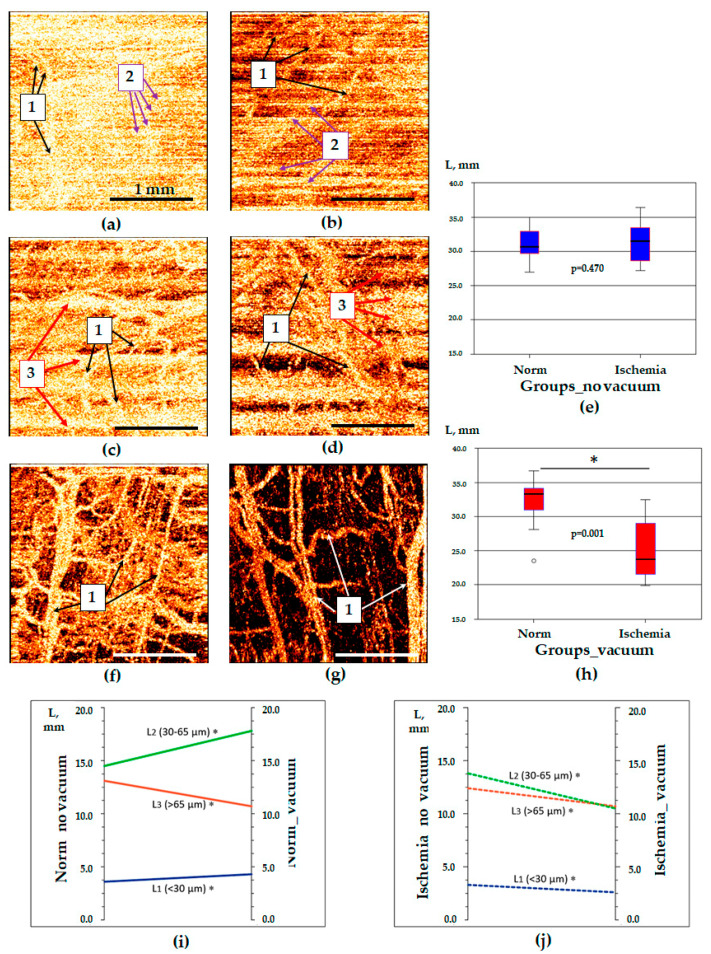
OCTA images of the small intestine obtained in open surgery and their quantification. (**a**,**c**,**f**)—normal intestine, (**b**,**d**,**g**)—ischemic intestine; (**a**–**d**)—manual pressure was used for tissue stabilization; f—tissue stabilization was achieved by developed vacuum unit; (**e**,**h**)—median values of the total blood vessels length in OCTA images of normal and ischemic intestine using manual fixation of the probe (**h**) and developed vacuum unit (**h**); (**i**)—changes in the lengths of various-diameter vessels in OCTA images of normal intestine with mechanical fixation (norm_no_vacuum) and using vacuum fixation (norm_vacuum); (**j**)—changes in the lengths of various-diameter vessels in OCTA images of ischemic intestine with mechanical fixation (ischemia_no vacuum) and using vacuum fixation (ischemia_vacuum). 1—blood vessels, 2—artifacts caused by mechanical probe vibrations, 3—artifacts caused by respiratory movements and peristalsis. *—Statistically significant differences in the length of blood vessels of a certain diameter in OCTA images due to the use of a vacuum stabilizer (**i**,**j**).

The use of the vacuum stabilizer led to a significant reduction in the proportion of non-informative OCTA images in both the normal and ischemic conditions. The proportion of the OCTA images of the normal intestine of the 3rd class (with a large number of artifacts) decreased by seven times—from 112 to 16 images out of 200 (Χ^2^ = 105.88, *p* = 0.001). At the same time, the proportion of the OCTA images of classes 1 and 2 were equal (each was 92). Thus, the use of the vacuum stabilizer was accompanied by an increase in the proportion of informative images of classes 1–2 in the normal intestine by a factor of 2.1 and in the ischemic intestine by a factor of 3. In general, the proportion of non-informative OCTA images in all segments of the intestine decreased by 78%: from 248 to 54 out of 400 images (Χ^2^ = 200.2, *p* = 0.001). At the same time, the image quality was increased mainly due to a reduction in the number of motion artifacts in the form of bright longitudinal wide bands (artifacts of respiratory movements and peristalsis) (Figure 3c,d), as well as due to artifacts in the form of thin frequent bands (artifacts of mechanical probe vibrations) (Figure 3a,b).

The median values of the total length of blood vessels (“L” parameter) in the studied groups were 33.3 [31.0; 34.1] mm in norm with vacuum stabilization, 31.5 [28.6; 33.5] mm in ischemia without vacuum stabilization (*p* > 0.05) and 23.8 [21.7; 28.9] mm in ischemia with the application of vacuum stabilization (*p* < 0.05). A comparison of OCTA images obtained in the traditional way (manual tissue fixation by probe) did not show statistically significant differences in the “L” parameter (Wilcoxon test, *p* = 0.470) between the normal and ischemic intestine (Figure 3e). A significant part of motion artifacts looks like the optical equivalent of blood vessels and may affect the results of quantification. In OCTA for images obtained using vacuum stabilization which enabled the elimination of most of the artifacts, the “L” parameter in the ischemic intestine was statistically significantly different from the normal one (Wilcoxon test, *p* = 0.001) (Figure 3h). To summarize, the vacuum unit utilization enabled a significant increase in the information value of the OCTA method, made it convenient for surgeons, and significantly reduced the time of diagnostics.

In the experiment series 1 (minipigs, open surgery), the analysis of blood vessels of various diameters in OCTA images showed that in the normal intestine, vacuum stabilization was accompanied by an increase in the length of optical objects identified as blood vessels of small (*p* = 0.003) and medium (*p* = 0.001) diameters, while the length of blood vessels with a diameter of more than 65 µm was decreased (*p* = 0.001) (Figure 3i and Table 2). In the ischemic intestine, an increase in the image quality after using a vacuum stabilizer was accompanied by a proportional reduction in the length of optical objects recognized as the vessels of the small (*p* = 0.008), medium (*p* = 0.004) and large (*p* = 0.002) diameters (Figure 3j and Table 2). Considering the qualitative changes in the OCTA images, the described changes occurred due to the reduction in the number of motion artifacts, the bright lines of which were recognized as vessels of large diameter in the normal intestine and as vessels of large, medium and small diameters in the ischemic state.

### 3.2. OCTA Image Quality in Laparoscopic Surgery

OCTA images obtained in laparoscopy contained a greater number of motion artifacts than those obtained in open surgery. For the manually controlled contact of the OCTA probe and the tissue surface, artefact-free OCTA images of class 1 were obtained in 12 observations out of 200; OCTA images with a small number of artifacts (grade 2) accounted for 32 images out of 200. About 78% of OCTA images were uninformative due to the large number of motion artifacts. In the ischemic intestine, the total proportion of informative OCTA images of classes 1 and 2 in laparoscopy did not exceed 28% (Table 3).

The use of the vacuum stabilizer during laparoscopic surgery led to a significant reduction in the proportion of non-informative OCTA images. However, their proportion was lower compared with the open surgery conditions. Using a vacuum stabilizer made it possible to increase the proportion of the OCTA images of the ischemic intestine of the 1st class from 2% to 14% and of the 2nd class—from 12% to 41%. However, non-informative OCTA images were obtained in 48% of all cases (Table 3). At the same time, the total proportion of non-informative images of normal and ischemic tissues decreased by 51%—from 328 to 160 images out of 400 (Χ^2^ = 148.3, *p* = 0.001).

**Figure 4:**
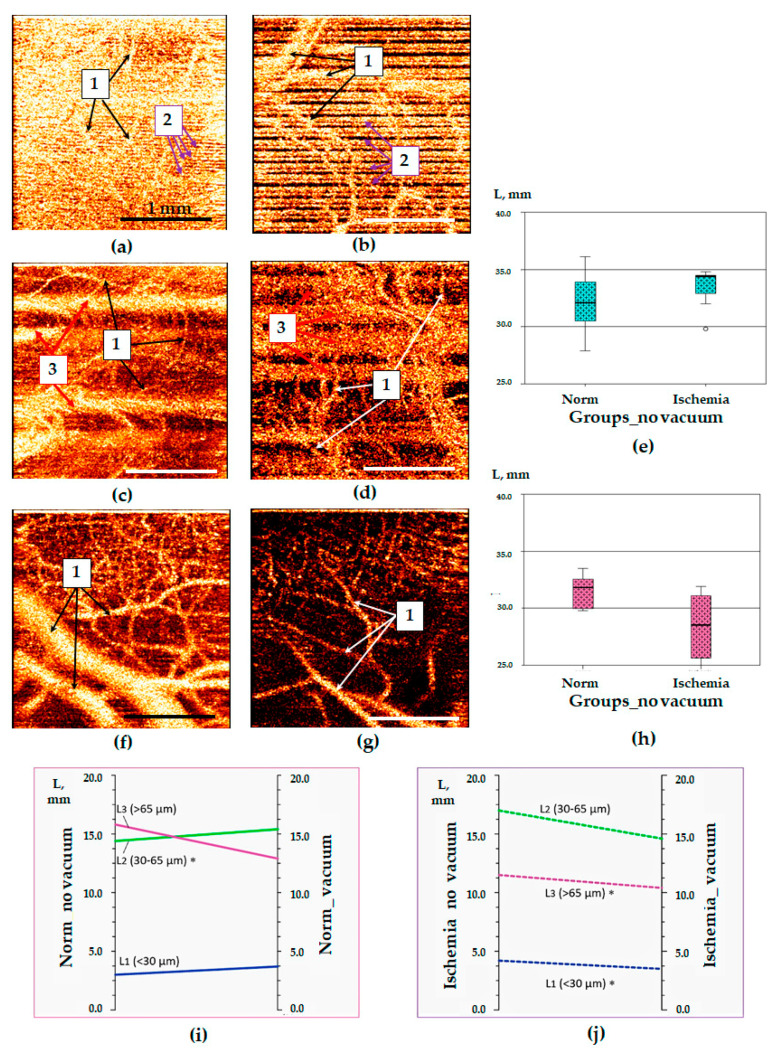
OCTA images of the small intestine obtained in laparoscopy and their quantification. Panels (**a**,**c**,**f**)—normal intestine; (**b**,**d**,**g**)—ischemic intestine; (**a**–**d**)—manually-produced pressure was used for tissue stabilization; (**f**)—tissue stabilization was achieved by the vacuum unit; (**e**,**h**)—median values of the total blood vessels length in OCTA images of normal and ischemic intestine using manual fixation of the probe (**h**) and developed vacuum unit (**h**); (**i**)—changes in the lengths of various-diameter vessels in OCTA images of normal intestine with mechanical fixation (norm_no_vacuum) and using vacuum fixation (norm_vacuum); (**j**)—changes in the lengths of various-diameter vessels in OCTA images of ischemic intestine with mechanical fixation (ischemia_no vacuum) and using vacuum fixation (ischemia_vacuum). 1—blood vessels, 2—artifacts caused by mechanical probe vibrations, 3—artifacts caused by respiratory movements and peristalsis. *—Statistically significant differences in the length of blood vessels of a certain diameter in OCTA images after the use of a vacuum stabilizer (**i**,**j**).

The improvement in the quality of OCTA images when using a vacuum stabilizer (Figure 4c,f) was achieved by reducing the number of motion artifacts caused both by mechanical probe vibrations (Figure 4a,b) and the peristaltic wave and transmission vibrations from diaphragm movements (Figure 4c,d). The second type of artifact was present on most OCTA images of both intact and ischemic intestines in the form of 3–4 light inhomogeneous bands with fuzzy contours (Figure 4c,d). The frequency of motion artifacts depended on the depth of ischemic damage: in the early stages of ischemia, motion artifacts were most pronounced; with deep damage after the development of paresis of the intestinal wall, the number of motion artifacts was reduced. However, in general, the proportion of non-informative OCTA images with a large number of artifacts in the ischemic areas of the intestine was greater than in the normal intestine (Figure 3a,b).

The comparison of the length of perfused blood vessels in the OCTA images for rabbits in laparoscopy showed the same trend as during open surgery for minipigs (Figure 3). The median value of the “L” parameter in the normal intestine without the use of the vacuum stabilizer was 32.1 [29.0; 33.8] mm and in the ischemic intestine without the stabilizer it was 34.4 [32.0; 34.5] mm (*p* = 0.345). With the use of the tissue stabilizer, median values of the “L” parameter differed more pronouncedly: in the normal intestine it was 31.8 [29.9; 33.1] mm, in the ischemic intestine—28.5 [25.3; 31.3] mm (*p* = 0.091).

The analysis of the blood vessel lengths dependingon their diameter in rabbits showed the same trends asin minipigs (Table 2). In particular, in a normalintestine, the use of the vacuum stabilizer led to astatistically significant (*p* = 0.025) increase in theblood vessels length of medium diameter (30–65 µm) (Table 2 and Figure 4i). After vacuum stabilization inthe ischemic intestine, the length of optical objectsrecognized as the blood vessels of the small and largerdiameter demonstrated a statistically significantdecrease (with *p* = 0.043 and *p* = 0.034, respectively) (Figure 4j). Similar changes in the length of theoptical equivalents of medium diameter blood vesselswere not recorded (*p* = 0.128) (Figure 4j). The visualassessment of the OCTA images showed that thesechanges were not due to the reduction in the length ofthe perfused vessels, but caused by the elimination ofthe motion artifacts (strips of various thicknesses), which partly overlapped contours of blood vessels and partly imitated them (Figure 4b,d,g).

### 3.3. Safety of the Vacuum Stabilization Method for Intestinal Wall Tissues

The pressure deficit in the lumen of the vacuum unit was maintained using an operating aspirator. In open surgery in minipigs, MAP fluctuated at the level of 85 [77; 91] mm Hg; IAP was equal to atmospheric pressure. Therefore, the pressure deficit in the lumen of the vacuum unit was maintained at a level of 25 mm Hg. In laparoscopic surgery in rabbits, when calculating a safe level of pressure deficit, it was taken into account that IAP was greater than atmospheric pressure. IAP created during insufflation into the abdominal cavity of gas was 10 [8.5; 11] mm Hg; MAP fluctuated at the level of 92 [85; 94] mm Hg. To maintain the PP at a level of at least 60 mm Hg, the pressure deficit in the device was maintained at a level of ≤22 mm Hg. 

**Figure 5:**
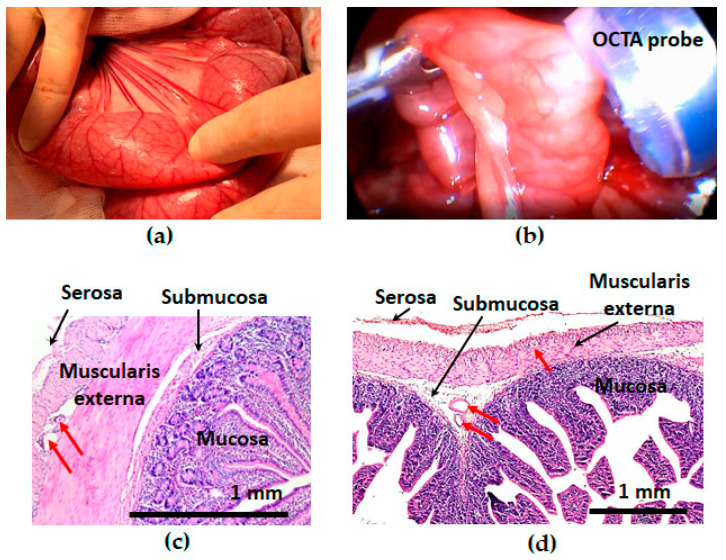
Macroscopic (**a**,**b**) and histological (**c**,**d**) picture of the intramural circulation and intestinal tissues after the use of the vacuum stabilization unit in open abdomen surgery (**a**,**c**) and laparoscopy (**b**,**d**). Red arrows indicated empty blood vessels; no signs of circulatory disorders were detected.

The macroscopic picture of the state of the intestinal wall while observing the parameters of a safe pressure deficit in the vacuum stabilization unit did not differ from the normal one. Hematomas, ruptures, and other injuries were not recorded either in open abdomen surgery or laparoscopy. As a rule, after the removal of the device, rounded areas of hyperemia remained on the surface of the intestine (Figure 5a,b). Within 1–5 min, the trace of hyperemia on the intestinal wall spontaneously disappeared. A histological examination of the intestinal wall obtained from the area of application of the device confirmed the absence of pathological changes in all cases (Figure 5c,d).

## 4. Discussion

The quality of images of the microvasculature is influenced by several groups of factors. Some of them depend on the actions of the operator and the characteristics of the device [38], other factors relate to the patient’s condition—the blood pressure or degree of sedation [39,40]. As a result, 20 to 80% of images become uninformative. Pressure and motion factors are of the greatest importance for the quality of high-resolution bioimaging data [3,37,41]. The physiological motions of the object during the study lead to the appearance of motion artifacts and critically reduce the information content of images. However, any attempts to stabilize the tissues with the operator’s mechanical pressing cause pressure artifacts, which also reduce the quality of the data obtained.

High resolution bioimaging also requires tissue immobilization and in this case devices of various designs with a universal mechanism of vacuum stabilization are often used [6,33]. An important advantage of the vacuum unit described in this paper is the simplification of the intraoperative study: there is no more need of the fixed static position of the operator, which is necessary in the traditional method of mechanical pressing to prevent the movement of the probe relative to tissues. Our results showed that various movements of the operator’s hands during the probe holding, as well as physiological motions of the studied tissues, did not lead to the displacement of the tissues relative to the working surface of the probe and did not affect the quality of the OCTA images.

In laparoscopy or robotic surgery, when the direct control of the OCTA probe by the operator’s hands is impossible, the use of stabilizers that temporarily immobilize the tissue becomes especially important [42]. In addition, the stabilizing devices acquire the function of an additional manipulator. It is important to ensure that the applied vacuum level is safe: after its application, hematomas, ischemic disorders or tissue micro-ruptures should not appear. We have developed and tested an approach in which the safe level of pressure deficit in the stabilizer is calculated based on the MAP and IAP levels and is limited to a known safe level of PP. Therefore, it seems physiologically justified and practically accessible.

The study has some limitations that should be mentioned. Some of the artefacts in OCTA images of the intestine are not associated with bowel movements and cannot be reduced using the developed unit. For example, artefacts caused by changes in blood pressure [35]. The complete leveling of artifacts is possible only after the development of comprehensive measures that take into account all significant pathophysiological mechanisms of image distortion. In addition, a small amount of clinical data obtained during a real intraoperative examination of patients has not yet allowed us to collect the necessary amount of data for statistical processing. The conditions under which the data obtained during the examination of laboratory animals are as close as possible to the real conditions of the clinic (including drugs for anesthesia, hemodynamic control devices, surgical instruments, etc.), but they cannot be completely identical. Further intraoperative studies in large laboratory animals and in real clinical settings are required to obtain conclusive results.

Prospects for the clinical use of OCTA in bowel surgery are associated with diagnostic and therapeutic tasks. Diagnostic direction is one of the most important: OCTA can become the leading method for diagnosing intramural blood and lymphatic vessels, lymphoid tissue [43]; in addition, using OCT elastography [44], it is possible to assess inflammatory changes in the intestine [45]. Another promising direction for clinical OCTA implementation is the provision of microsurgical interventions under OCTA navigation: the removal of tumors, polyps, a biopsy, and the imposition of micro-surgical anastomoses. Most operations and diagnostic manipulations on the intestines are now performed under laparoscopic control. The number of robotic operations on the intestines is growing rapidly. To increase the autonomy and accuracy of these operations, 3D navigation systems with the possibility of using microsurgical interventions are needed [46,47]. The OCTA technology with the fixation of the examined part of the intestine can become an optimal tool not only for improving the quality of images, but also for creating conditions for precise microsurgical interventions under OCTA navigation. However, to realize this potential, it is necessary to integrate the OCTA technology—mechanical, software components—into existing and future laparoscopic complexes and robotic surgery complexes.

An additional potential way to overcome motion artifacts in OCTA may be the use of convolutional neural networks (CNN) to visualize vessels from single B-scans. While several of such approaches have been proposed in recent years [48,49,50,51], they are yet to be applied to the organs of humans and other big animals other than the eye in clinical settings. Two major problems have to be outlined here. Firstly, while the eye profiles, created in the OCT B-scans by the blood vessels are clearly visible, it is not the case in other human organs, which is especially true for the smaller vessels. Secondly, the successful training of the CNN requires a rather big datasets, the collection of which is much easier for well-established medical investigation, such as eye visualization with ophthalmoscopic OCT [52], or for the wide-spread small-animal models [49,50,51]. The extensibility of the CNNs trained on such datasets to other localizations is still an open question.

## 5. Conclusions

The results of OCTA visualization of blood circulation in the small intestines of laboratory animals (rabbits and minipigs) showed that without the stabilization, OCTA images are uninformative in 56–68% of cases in open surgery and in 78–86% of cases in laparoscopy. Notably, the OCTA study of the ischemic intestine wall is associated with a greater number of artefacts than the study of the normal one (*p* = 0.030).

The use of a vacuum stabilization unit with a pressure deficit of 22–25 mm Hg during OCTA allowed for the significant reduction of the number of artefacts caused by the movement of tissues relative to the probe (respiratory, pulse, muscle contractions). As a result, the proportion of non-informative OCTA images in open surgery decreased by 78.0% (Χ^2^ = 200.2, *p* = 0.001), and the proportion of high-quality OCTA images of grades 1–2 become 86.5%. In laparoscopy, there was the same tendency: a significant reduction in non-informative OCTA images (Χ^2^ = 148.3, *p* = 0.001) and an increase in the proportion of high-quality artefact-free OCTA images up to 60% were observed.

The results obtained largely remove the obstacles to the widespread introduction of OCTA technology into the clinic for the diagnosis of acute intestinal ischemia. To further realize the diagnostic potential of OCTA in abdominal surgery, it is necessary to integrate the technology into existing laparoscopic and robotic surgery complexes.

## Figures and Tables

**Table 1:** The number of OCTA images of the small intestine (minipigs) of classes 1, 2 and 3 according to Magnin M. et al. (2020) [37] in the conditions of the “open abdomen”.

Group, Stage of Experiment	Image Quality Class					
	1 Class		2 Class		3 Class	
	MP *	VS **	MP	VS	MP	VS
Norma (n = 200), n	14 (7%)	92 (46%)	74 (37%)	92 (46%)	112 (56%)	16 (8%)
Ischemia (n = 200), n	8 (4%)	48 (24%)	56 (28%)	114 (57%)	136 (68%)	38 (19%)

*—number of OCTA images obtained using mechanical pressing (MP) of the probe to the tissues; **—number of OCTA images obtained using developed unit—vacuum stabilizer (VS).

**Table 2:** Dynamics of the length of blood vessels of different diameters (L1 for d < 30 µm, L2 for d = 30–65 µm, and L3 for d > 65 µm) in OCTA images of normal and ischemic intestines after the use of a vacuum stabilizer, Me [Q_1_;Q_3_].

	Normal Intestine, Experiment Series 1			Ischemic Intestine, Experiment Series 1			Normal Intestine, Experiment Series 2			Ischemic Intestine, Experiment Series 2		
Vessel diameter	L1	L2	L3	L1	L2	L3	L1	L2	L3	L1	L2	L3
No vacuum	3.6 [3.1; 4.2]	14.5 [12.5; 16.1]	13.1 [12.1; 14.3]	3.3 [3.2; 4.4]	13.8 [12.7; 17.2]	12.4 [12.2; 13.9]	3.0 [2.9; 3.6]	14.4 [13.2; 14.7]	15.8 [12.9; 15.9]	4.2 [4.1; 4.8]	17.0 [16.3; 17.5]	12.4 [12.2; 13.9]
Vacuum stabilizer	4.3 [3.9; 4.6]	17.8 [16.5; 19.5]	10.7 [9.7; 11.5]	2.6 [2.0; 3.4]	10.5 [8.7; 13.8]	10.7 [10.2; 11.3]	3.7 [3.6; 3.9]	15.4 [15.3; 16.0]	12.9 [11.2; 13.5]	3.5 [2.8; 4.0]	14.6 [12,1; 16.0]	10.4 [9.9; 10.8]
Wilcoxon signed-rank test	0.003	0.001	0.001	0.008	0.004	0.002	0.239	0.025	0.138	0.043	0.128	0.034

**Table 3:** The number of OCTA images of the small intestine (rabbits) of classes 1, 2 and 3 according to Magnin M. et al. (2020) [37] in laparoscopy.

Group, Stage of Experiment	Image Quality Class					
	1 Class		2 Class		3 Class	
	MP *	VS **	MP	VS	MP	VS
Norma (n = 200), n	12 (6%)	42 (21%)	32 (16%)	88 (44%)	156 (78%)	70 (35%)
Ischemia (n = 200), n	4 (2%)	28 (14%)	24 (12%)	82 (41%)	172 (86%)	90 (45%)

*—number of OCTA images obtained using mechanical pressing (MP) of the probe to the tissues; **—number of OCTA images obtained using developed unit—vacuum stabilizer (VS).

## Data Availability

Not applicable.
